# Exploring needs, prevalence and experience with robotic-assisted surgery training among residents: a mixed method study

**DOI:** 10.1007/s11701-025-02527-7

**Published:** 2025-07-15

**Authors:** M. Marije Zwakman, M. Miranda Trippenzee, J. F. M. Johan Lange, J. P. E. N. Jean-Pierre Pierie, E. C. J. Esther Consten

**Affiliations:** 1 Department of Surgery, University Medical Center Groningen, Hanzeplein 1, 9713 GZ Groningen, The Netherlands; 2 Department of Health Psychology, Health Sciences, University of Groningen and University Medical Center, Groningen, The Netherlands; 3 Department of Surgery, Medical Centre Leeuwarden, Henri Dunantweg 2, 8934 AD Leeuwarden, The Netherlands; 4 Postgraduate School of Medicine, University Medical Center Groningen, Hanzeplein 1, 9713 GZ Groningen, The Netherlands

**Keywords:** Robotic assisted surgery, Surgical education, Residency training, Mixed methods research, Needs assessment

## Abstract

Robotic-assisted surgery (RAS) is increasingly used in clinical practice, but training opportunities for residents vary widely. This multicenter, mixed-methods study assessed Dutch residents’ exposure to RAS, identified their training needs, and explored their perspectives on the future of RAS through a survey (*n* = 148) and groups interviews (*n* = 20). Informed consent was obtained. While 69.6% of the residents had RAS experience, only 14.2% had operated as console surgeon. Despite robots being available in 75.7% of hospitals, hands-on training remained limited. Most residents (77.7%) expressed interest in a national RAS curriculum. Qualitative data revealed four major themes: needs and experiences, training requirements, the role of the OR team and industry, and assessment and feedback, with residents advocating for early exposure and holistic feedback. There is a clear need for a formalized RAS training program. Future research should focus on structured curricula, tailored to resident needs, including possible barriers and facilitators.

**Supplementary Information:**

The online version contains supplementary material available at 10.1007/s11701-025-02527-7.

## Introduction

The widespread adoption of robotic-assisted surgery (RAS) has rapidly expanded in recent years, with the Netherlands currently hosting 45 robots in 31 out of 68 hospitals [1, 2]. This number is continuously growing due to the increasing range of RAS applications and the emergence of new suppliers in the market. As previous literature has highlighted, RAS is poised to become an integral part of the future of surgical practice, as endorsed by program directors, staff and residents [3–6]. Consequently, there is a burgeoning demand for comprehensive training programs to equip residents across various specialties with the necessary RAS skills.

While validated, concise training programs, such as the DaVinci Technology Training Pathway, Fundamentals of Robotic Surgery, Fundamental Skills of Robot-Assisted Surgery, and the Robotics Training Network curriculum are worldwide available, RAS training courses are often industry-sponsored, costly, require extensive travel, and often lack the dedicated practice time that residents need [7–15]. Additionally, when both staff and residents are in the learning phase, residents may be temporarily excluded from participating in RAS procedures [16–20]. The unequal access to training programs poses a significant challenge for most residents, exacerbating the transition from training to independent practice [21].

This challenge is further compounded by the difficulty of integrating existing RAS training programs into specific residency curricula, due to variations in content, design, and their predominant focus on staff rather than residents [7–10]. Therefore, identifying residents’ needs and customizing training programs to attain desired outcomes is crucial. These programs should be tailored by understanding the primary end users: residents. A better understanding of their perspectives will facilitate the development of future training programs and enhance compliance with learning RAS. While previous literature has mainly focused on residents’ evaluations and opinions of training programs, the prerequisites for establishing a multidisciplinary RAS training program in the Netherlands have not been extensively explored [22–25].

The Dutch Health and Youth Care Inspectorate (IGJ) has emphasized the need for structured education in all forms of minimal invasive surgery, including the formulation of starting criteria and the measurement of capability and competency in all surgical specialties performing RAS [26, 27]. Given the absence of a nationwide, multidisciplinary RAS training program for residents at the time of this study, this research serves as a foundation.

The objective of this study is to assess the extent of current exposure that Dutch surgical residents have to RAS and RAS training. Additionally, the study will delineate the needs, requirements, and objectives for RAS training program development from Dutch residents in general surgery, gynecology & obstetrics and urology to provide a solid foundation to guide the development of a structured, nationally standardized RAS training curriculum that addresses residents’ specific requirements. This can potentially be extrapolated to other countries.

## Materials and methods

### Study design

This multicenter study was conducted in the Netherlands, encompassing all 488 surgical residents who were invited to complete a questionnaire. The questionnaire was distributed anonymously in accordance with privacy regulations concerning employee surveys. Simultaneously, the residents specializing in surgery, urology, and gynecology & obstetrics were invited to engage in group interviews. The qualitative design allows the residents to express their needs and preferences more extensively. Both quantitative and qualitative approaches were employed for data collection and analysis, constituting a ‘mixed methods’ approach. Due to the anonymous collection of quantitative responses, it was not possible to establish a link between those who completed the questionnaire and the residents invited to participate in the group interview.

### Primary and secondary outcomes

The primary outcome is surgical residents’ overall exposure and experience with RAS, their educational exposure and experience with RAS training, as well as their opinion on the importance of RAS for their future. The secondary outcomes encompass the assessment of preferences, needs and goals related to the development of an RAS training program for residents in general surgery, gynecology & obstetrics, and urology.

### Measurement of residents’ exposure to RAS and RAS training

An anonymous web-based survey, consisting of 24 multiple-choice and multi-answer questions, was designed to gain insights into current RAS experience, current training experience and perspectives on future practice. Data on the availability of a surgical robot, institutional information, individual participation in RAS training and robotic cases, and future perspectives were collected. The questionnaire was developed based on a literature review and expert input, identifying key themes in RAS training and ensuring content validity through internal review [4, 6, 21, 28–31]. A detailed and translated overview of the survey is presented in **Supplementary Table 1**.

The responses were collected over a 4-month period from October 2023 to January 2024. Email addresses of the secretaries overseeing resident program were procured. To ensure compliance with privacy legislations, these secretaries disseminated an email containing a survey link to all surgical residents. Additionally, the survey links were shared through mobile phone-based chat groups and national newsletters of the residents’ associations. Survey responses were collected and managed using RoQua, a secure, web-based software platform hosted at the University Medical Center Groningen. Data collection and storage adhered to the principles of the Declaration of Helsinki.

### Residents’ needs and preferences for RAS training

To explore the preferences, needs and goals of residents, a needs assessment was conducted during group meetings from February 2024 to April 2024. Each distinct group consisted of a mix of 6 to 7 surgical, urology, and gynecology & obstetrics residents, with a total of 20 participants. Recruitment of group participants occurred through two ways: at the end of the questionnaire, respondents were given the option to contact the researcher themselves if they were interested in participating in a group interview; and second an online invitation was distributed via educators and education coordinators, who were asked to forward it to residents. Informed consent was obtained from all participants.

Previous literature on perceptions from residents was used to develop an interview guide, see **Supplementary Table 2**. Questions were formulated to accompany each topic, which revolved around identifying the essential resources, services, or interventions needed for RAS training, to achieve residents’ specific goals. Interview questions were broad and applied to any form of RAS training, including e-learning, dry- and wet-lab, simulation and console training. The interview guide and formulated questions were reviewed and deliberated upon by three co-authors.

The interviews were moderated by one member of the research team (MZ). A second member of the research team made observations and took field notes (MT). All group interviews lasted approximately two hours, were voice-recorded and transcribed verbatim.

### Data analysis

Descriptive statistics were calculated for all available variables from the web-based survey. All statistical analyses were conducted using SPSS® Statistics version 28.0 (SPSS Inc., Chicago, IL, USA).

To explore the residents’ needs, transcribed data were analyzed and coded, using qualitative data analysis software Atlas.ti. The interpretation methods, such as thematic analysis or content analysis, were deemed most appropriate for this type of study. These methods support qualitative analysis while allowing for the quantification of data to identify common themes and issues [32]. Codes that emerged from the data were created throughout the analysis, and overarching themes were subsequently formed based on clusters of these codes. Two research team members [MZ and MT] coded independently to ensure variated interpretations. Differences in coding were discussed until agreement was reached. A third author [EC] was involved in the event of any discrepancies. The group interviews were conducted by researchers with complementary backgrounds: a surgical resident [MZ] familiar with the clinical context, and a social scientist, who is also a lecturer in professional development and has qualitative research expertise. Both researchers engaged in ongoing reflexive practice to acknowledge and mitigate potential biases related to their professional roles and views on industry involvement in surgical education. The research team included a social scientist [MT] to improve qualitative data interpretation and add a non-medical perspective to the analysis of the data.

Ethical approval was not required as the study involved voluntary participation without patient data.

## Results

In total, 148 surgical residents (30.3%) completed the web-based survey, see Table 1. All years of residency were approximately equally represented. Most residents (75.7%) reported that their hospitals had a surgical robot present, and the majority possesses the type Xi from Intuitive, although a number of hospitals have multiple types of surgical robots. When ‘other’ was specified, the Hugo system from Medtronic was most commonly (*n* = 2) identified as the alternative robotic platform, followed by Transenterix (*n* = 1), now known as Asensus Surgical. Two participants did not clarify which other system was used in their hospital. The dual console, which consists of two separate workstations and thereby allows a surgeon and a resident to work together during a procedure, is reported to be available in 42.6% of the respondent’s hospitals. However, almost 20% of the residents are unaware of the type of console that is available.

**Table 1:** Baseline characteristics and institutional information of robotic-assisted surgery in the Netherlands

	Surgical residents, *n* (%)
*Number of respondents*	148
*Year of residency*	
Year 1	20 (13.5)
Year 2	23 (15.5)
Year 3	21 (14.2)
Year 4	26 (17.6)
Year 5	29 (19.6)
Year 6	29 (19.6)
*Surgical robot present at hospital?*	
Yes	112 (75.7)
No	36 (24.3)
Which type of robot present at hospital?*	
Si	4 (3.4)
X	15 (12.9)
Xi	64 (55.2)
Other	5 (4.3)
Unknown	31 (26.7)
*Dual or solo console present at hospital?*	
Dual console	63 (42.6)
Solo console	29 (19.6)
Unknown	28 (18.9)
*Dedicated robot-team present at hospital?*	
Yes	105 (70.9)
No	42 (28.4)
Unknown	1 (0.7)

^*^In three hospitals different types of the Intuitive robot (e.g., X and XI) are present

### Residents’ exposure to RAS

As shown in Table 2, a substantial proportion of surgical residents (30.4%) have not yet gained any experience with RAS. Among those residents who do have experience with RAS, the majority (34.5%) indicates having functioned as a bedside assistant in 1–10 procedures. Additionally, 21.6% have operated as the second console surgeon in 1–10 procedures, while 10.1% have assumed the role of the first console surgeon in 1–10 procedures. A minority has conducted more than 50 procedures as the second console surgeon (2.7%), and a further 4.1% have engaged in 10–50 procedures as the first console surgeon. The initial exposure to RAS occurred for 18.9% prior to commencing their residency, for example as researcher. Subsequently, residents encountered RAS in their respective third or fourth year (both 12.8%).

**Table 2:** Robotic-assisted surgery experience among Dutch surgical residents

	Surgical residents, *n* (%)
*In what year was your first experience with robotic-assisted surgery?*	
No experience yet	45 (30.4)
Before residency	28 (18.9)
Year 1	15 (10.1)
Year 2	11 (7.4)
Year 3	19 (12.8)
Year 4	19 (12.8)
Year 5	8 (5.4)
Year 6	3 (2.0)
*Time since first experience with robotic-assisted surgery*	
Never	54 (36.5)
0–6 months	16 (10.8)
6–12 months	23 (15.5)
1–5 years	55 (37.2)
*Experience as bed-side assistant*	
Never	67 (45.3)
1–10 procedures	51 (34.5)
10–50 procedures	26 (17.6)
> 50 procedures	4 (2.7)
*Experience as first console surgeon*	
Never	127 (85.8)
1–10 procedures	15 (10.1)
10–50 procedures	6 (4.1)
> 50 procedures	0 (0)
*Experience as second console surgeon*	
Never	103 (69.6)
1–10 procedures	32 (21.6)
10–50 procedures	9 (6.1)
> 50 procedures	4 (2.7)

### Residents’ exposure to RAS training

Reported experience with RAS-training is low, see Table 3. The majority of the participants (60.1%) did not used the online module of Intuitive, did not participate in in-service training of Intuitive (79.1%), in training at their own operation room (84.5%) or training in a wet-lab (85.1%) in preparation for RAS. Nonetheless, interest in RAS-training is high; 77.7% of the residents express interest in participating in a nationally organized training. Residents recommend commencing RAS-training in the third year (33.1%) or fourth year (30.4%). The robotic skills simulator was used more frequently after the first participation as bed-side assistant (17.6% vs. 28.4%) but used less after the first participation as a console surgeon (22.3% vs. 18.2%).

**Table 3:** Robotic-assisted training experience among Dutch surgical residents

	Surgical residents, *n* (%)
*Did you used the online module of Intuitive?*	
Yes, and completed	40 (27)
Yes, but not completed	19 (12.8)
No	89 (60.1)
*Did you participate in the in-service training of Intuitive?*	
Yes	31 (20.9)
No	117 (79.1)
*Did you participate in a training at your own operation room?*	
Yes	23 (15.5)
No	125 (84.5)
*Did you participate in a wet-lab training, for example at the ORSI Academy?*	
Yes	22 (14.9)
No	126 (85.1)
*Did you used the Robotic skills simulator before participating as bed-side assistant?*	
Yes	26 (17.6)
No	56 (37.8)
N.a	66 (44.6)
*Did you use the Robotic skills simulator after your first participation as bed-side assistant?*	
Yes	42 (28.4)
No	40 (27.0)
N.a	66 (44.6)
*Did you used the Robotic skills simulator before participating as console surgeon?*	
Yes	33 (22.3)
No	14 (9.5)
N.a	101 (68.2)
*Did you use the Robotic skills simulator after your first participation as console surgeon?*	
Yes	27 (18.2)
No	20 (13.5)
N.a	101 (68.7)
*In which year of residency would you implement robotic-assisted training?*	
Year 1	16 (10.8)
Year 2	14 (9.5)
Year 3	49 (33.1)
Year 4	45(30.4)
Year 5	19 (12.8)
Year 6	4 (2.7)
Fellowship	1 (0.7)
*Would you participate in a national-organized robotic-assisted training?*	
Yes	115 (77.7)
No, I already participated in a national-organized robotic assisted training	3 (2.0)
No, I am not interested	30 (20.3)

### Residents’ future perspectives

Data, as presented in Table 4, show that residents anticipate the selective utilization of RAS alongside laparoscopy (76.4%). A mere 1.4% of the respondents envision the obsolescence of RAS, while 6.1% consider it to become the new ‘golden standard’. Perceptions regarding the incorporation of RAS within their residency programs vary; 44.6% anticipate its limited utilization, whereas 27.7% believe it will likely not be integrated, with an additional 14.2% expressing the view that it will absolutely not be incorporated.

**Table 4:** Resident’s future perspectives of using robotic-assisted surgery

	Residents, *n* (%)
*How important will robotic-assisted surgery be in the future?*	
Robotic-assisted surgery will disappear	2 (1.4)
Robotic-assisted surgery will be used selective, next to laparoscopy	113 (76.4)
Robotic-assisted surgery will take over most of the laparoscopic procedures	24 (16.2)
Robotic-assisted surgery will be the new ‘golden standard’	9 (6.1)
*Do you think you will use the robot during your residency program?*	
Yes, in abundance	20 (13.5)
Yes, but limited	66 (44.6)
No, probably not	41 (27.7)
No, absolutely not	21 (14.2)
*Do you think you will use the robot after graduating from residency?*	
Yes, in abundance	18 (12.2)
Yes, but limited	69 (46.6)
No, probably not	44 (29.7)
No, absolutely not	17 (11.5)

### Residents’ needs and perspectives

Data from the group interviews revealed four major themes: needs and experiences, training requirements, role of the OR team and industry, and assessment and feedback.

### Needs and experiences

Residents express a clear demand for early and structured involvement in RAS during their residency. Their experience with RAS varies, with some residents reporting ample opportunities and support for training, while others perceive limited encouragement from trainers and consultants. This disparity is often linked to the fact that some consultants are still in the process of mastering robotic-assisted surgery, or the absence of a consensus on the use of robotic systems for all types of surgeries.

*"I had the advantage that one of the robotic gynecologists at the hospital where I worked was a proctor himself and was simply the most experienced person there."* – GYN2.

Others face challenges due to the exclusive use of these systems by consultants or fellows. As a result, some residents feel excluded, even in hospitals where the equipment is available. The residents also note that in some hospitals, the robotic systems are only accessible outside working hours, which is seen as an advantage by some, but as a disadvantage by others.

*"…because, you can train, but it always has to be during your shift, in the evening, or on the weekend—if the robot is not in use at that time."* – SURG3.

Many residents suggest that robotic training should be more widely available across surgical specialties, as robotic systems are seen as the future of surgery, and limiting access could hinder their professional development.

Additionally, residents note a variability with RAS training depending on the hospital and region they are in. While some hospitals provide opportunities for training, others do not, making the availability of robots a critical requirement for practice. There is a strong call for a standardized national training program that fosters inter-hospital and interdisciplinary collaboration, ensuring that residents, regardless of their specialty or region, have the opportunity to practice RAS. Centralization of healthcare increases the importance of such a program, as some residents have had to seek training opportunities at other hospitals due to the unavailability of robotic systems in their own settings. Furthermore, many residents rotate between different hospitals during their training, and the lack of access to robotic systems at some sites exacerbates the disparities in training opportunities.

*“I don’t know if similar developments are happening in other regions, but prostate cancer care in region X is highly centralized. They have decided that prostatectomies, which are performed using the robot, will be done in one centre by a dedicated group of urologists. And in order to ensure quality, here’s the catch, no residents are present during these procedures.”* – URO4.

### Training requirements

For effective RAS training, residents emphasize the need for a well-structured curriculum, trained staff, and appropriate equipment, including access to robots and diverse training facilities. The training should ideally begin in the first or second year of residency, with initial exposure to robotic systems through e-learning, bed-side assistance and simulation training, while procedural training through wet-labs should be introduced in the last two to three years of residency. Some residents suggested that a dual console system could enhance safety and supervision during training, enabling supervisors to guide residents more effectively. However, others raised concerns that this setup could hinder residents’ learning, as supervisors can easily take back control, potentially limiting hands-on experience.

The curriculum should be modular and stepwise, incorporating both technical and non-technical skills. There is a preference for training in error management, potentially through video-based learning.

*"But we record things and review them afterward. I can imagine that it’s surprisingly educational to watch your own procedure and reflect on how you could have done it more efficiently."* – GYN4.

Some of the residents highlight the importance of communication and situational awareness during console operations. However, residents also point out that many non-technical skills are covered in other parts of their training and do not require robotic-specific focus.

*"…maintaining communication with the OR team, while you’re actually inside that thing."* – SURG8.

### Role of the OR team and industry

The operating room (OR) team plays a crucial role in resident training. Dedicated OR teams can provide valuable learning opportunities in handling the robot, assisting with robotic arms, and communicating during procedures. Trust between residents and the OR team is essential, especially since non-verbal communication is limited when the resident operates from within the robotic console. Operating room nurses, in particular, are seen as instrumental in bedside teaching and enhancing communication during RAS.

*“Yes, what is truly important is that the operating room nurse is many times more crucial in a robotic procedure than in an average other procedure […] and they can teach you how bedside teaching works, much better than the average surgeons, I believe.”* -SURG8.

The role of the medical industry in RAS is viewed as minimal by residents, though they acknowledge that industry representatives are helpful in the early familiarization stages with the robotic system.

### Assessment and feedback

Although there is no widespread call for formal assessments, residents appreciate the idea of earning a diploma or certification to validate their participation in RAS training. Methods such as OSATS (Objective Structured Assessment of Technical Skills) or Entrustable Professional Activities (EPA) could be used to provide structured feedback. However, residents also emphasize that assessments should not become the sole focus; they argue against the implementation of checklists that could serve as gatekeeping mechanisms for progressing to the next training stage. Instead, they advocate for a more holistic approach to evaluation that fosters learning rather than merely ticking off requirements. Additionally, residents express a desire for case logs and video recordings of their procedures for post-surgery review and group feedback sessions, which would support collective learning.

*“You consider with the trainer, is it good enough? Does the robot make it significantly different? Personally, I would appreciate eventually receiving some kind of certificate to demonstrate that I have completed the training […] I wouldn’t want to add a completely separate OSATS thing…”* – SURG1.

## Discussion

This study examined the current exposure and needs of Dutch residents in RAS training across general surgery, urology, and gynecology & obstetrics. It highlights a significant variability in access and experience, alongside a strong interest among residents in participating in structured, nationally organized RAS training programs. While consistent with prior international reports, our study contributes new insights by addressing the needs and preferences of residents across specialties through in-depth, qualitatively derived data. Our findings suggests an urgent need to formalize a RAS training curriculum within residency programs, and its success will depend on how well it addresses their specific needs.

Several key findings stand out in this study. First, 45.3% of residents have never acted as a bedside assistant in RAS, and 85.8% have never served as the first console surgeon, demonstrating limited hands-on experience. This aligns with previous literature and may reflect that supervising surgeons are still navigating their own learning curves [16–20, 44].

Additionally, RAS training in the Netherlands remains highly context-specific, depending on factors such as the particular hospital or region, specialty, the types of surgeries performed and the type of robot used. Recent literature confirms that nearly every hospital and specialty tends to develop its own individual training program [33–47]. However, our qualitative findings reveal that residents are not seeking further local customization, but rather express a strong desire for a nationally, standardized, stepwise RAS training program that can be extrapolated across hospitals and specialties. 77.7% of residents expressed interest in such a formalized pathway. They emphasized the importance of a modular structure that introduces robotic surgery gradually, beginning with basic exposure through e-learning, bed-side assistance, and simulation training in early training years. This phased approach was seen as crucial for building both technical and non-technical skills in a controlled, supportive environment. Procedural training, such as hands-on experience through wet-labs, was suggested to be introduced in the later years. This stepwise approach is consistent with previous developed RAS training programs [1, 22, 36–38].

Notably, residents prioritize structure and access over new forms of assessment. Some residents did suggest certification or Entrustable Professional Activity, supported by the common practice of using Objective Structured Assessment of Technical Skill, as proof of completed RAS training. The use of other internationally established tools—such as GEARs, R-OSATS, NASA-TLX, or GOALS—was not encouraged by the residents, despite their frequent application in existing RAS training programs [47, 40–51]. This option could be explored in future studies, in a barrier and facilitator analysis.

Furthermore, our qualitative data support previous observations; urology is currently the largest specialty utilizing RAS, whereas gynecology remains uncertain about its application, as it is not yet clear whether it offers significant benefits for patients [44, 45].

Despite the widespread presence of robotic systems in hospitals, including dual consoles in 42.6%, substantial gaps between equipment availability and training opportunities remain. This study reveals that a considerable proportion of residents lack access to essential training tools, with only 14.9% having participated in wet-lab training. These concerns are also described internationally, although access to RAS training appears to be more widespread in America [46, 47].

Another interesting finding of the study was the crucial role of the operating room (OR) team in supporting resident training in (RAS). The residents emphasized the importance of trust, especially given the limited non-verbal communication during robotic procedures, and encouragement provided by the team. Although the importance of communication with the OR team and a dedicated team are described before as essential, no literature was found on the role of the OR team in educating residents [48, 49].

Finally, residents expressed concerns about long-term relevance of robotic-assisted surgery training for their future practice. Some feared that RAS training may not apply to their eventual subspecialty, making it challenging to justify the time and resources invested. However, they acknowledged that basic familiarity with robotic systems could still be beneficial, as it may become a core competency for future surgeons, urologists, or gynecologists. While existing literature often focuses on subspecialties such as colorectal surgery or robot-urology, this study uniquely demonstrates that residents across specialties express a clear need for basic RAS training during their residency [24, 33, 35, 43, 50].

A key strength of this study is its mixed-methods approach, incorporating both quantitative survey data and qualitative group discussions. This methodology provides a nuanced understanding of residents’ experiences and needs, as the group discussions offer diverse perspectives on specific training requirements. While a survey could effectively gather information on needs, the group interviews highlight the varying levels of commitment among residents regarding RAS training. Furthermore, such studies are rarely conducted, making this research particularly valuable for gaining in-depth insights into the perceptions of residents.

However, there are limitations to consider. The response rate of 30.3% could introduce selection bias, as residents with stronger preferences on RAS may have been more likely to participate. Although this study has a low response rate, it aligns with the average response rate in surveys among surgical residents, which typically falls below 30 [51]. Additionally, the survey was only conducted among surgical residents, potentially limiting the generalizability of findings. The study is also limited by its reliance on self-reported data, which may not accurately reflect actual clinical exposure or training experiences. Furthermore, the group discussions were conducted online, which may have restricted the spontaneity of responses and led to more structured interactions. While there was a risk of participants echoing each other’s sentiments, it is important to note that our data also revealed instances of disagreement among participants, indicating a diversity of perspectives.

When viewed through the lens of curriculum development theory, this study primarily represents a targeted needs assessment—the second step in Kern’s six-step approach to curriculum development. Future research should build on these findings by defining clear educational objectives (step 3), followed by the developing educational strategies (step 4). These may include blended learning approaches combining e-learning, simulation, bedside mentoring, and hands-on wet-lab experiences. Steps 5 and 6 involve implementing and evaluating a national, interdisciplinary RAS curriculum, guided by a stakeholder analysis to identify and understand the key players involved, their interests, and the impact they may have on the success of such a training program.

Furthermore, a barrier and facilitator analyses may help to identify challenges in implementation and sustainability of the curriculum. Such analysis should specifically explore the discrepancy between residents’ needs and the current training infrastructure, including limited time allocation for RAS training, insufficient access to dual consoles, the absence of a structured national program, and the heavy dependence on local hospital settings or specialties. Understanding these barriers will be essential for designing a feasible and widely applicable training curriculum. To support future implementation efforts, a concise overview of key recommendations and considerations for national RAS curriculum development is provided in **Appendix A**.

In conclusion, the findings from this study highlight the pressing need for structured and accessible RAS training programs for Dutch residents. Despite the growing prevalence of robot systems in hospitals, significant barriers to training remain, including limited access to hands-on experience and inconsistencies in available training resources. Addressing these gaps through national, standardized curricula will be critical in ensuring that residents across all specialties are adequately prepared to meet the demands of modern surgical practice.

## Supplementary Information

Below is the link to the electronic supplementary material.

Supplementary file1 (DOCX 20 KB)

Supplementary file2 (DOCX 14 KB)

## Data Availability

Data are available upon reasonable request.
