# Supplementary material for: Exploring needs, prevalence and experience with robotic-assisted surgery training among residents: a mixed method study
Source: J Robot Surg. 2025 Jul 15;19(1):392. doi: 10.1007/s11701-025-02527-7 (PMC12263794; doi:10.1007/s11701-025-02527-7)
Supplement: Supplementary file 1 — Supplementary file1 (DOCX 20 KB) [file 11701_2025_2527_MOESM1_ESM.docx]

**Supplementary Table 1** Detailed overview of the survey, presented in English, translated from Dutch

|  | **Question** | **Answer options** |
| --- | --- | --- |
| Question 1 | Which year of residency are you in? | 1. Year 1 2. Year 2 3. Year 3 4. Year 4 5. Year 5 6. Year 6 |
| Question 2 | Does your hospital have a robot? | 1. Yes 2. No 3. Unknown |
| Question 3 | Do you know what type of robot is used in your hospital? | 1. Si 2. X 3. Xi 4. Other: […] 5. Unknown |
| Question 4 | Which console setup is used in your hospital? | 1. Solo console 2. Dual console 3. Other: […] 4. Unknown |
| Question 5 | Is there a dedicated robot OR team in your hospital? | 1. Yes 2. No 3. Unknown |
| Question 6 | Have you ever viewed an online module on the Intuitive/Da Vinci website? | 1. No 2. Yes, only viewed but not completed 3. Yes, and completed |
| Question 7 | Have you completed an in-service training delivered by an Intuitive representative? | 1. Yes 2. No |
| Question 8 | Have you followed training in your own OR, given by, for example, trained OR staff? | 1. Yes 2. No |
| Question 9 | Have you followed a wet-lab training (on pigs), as given in Orsi/Strassburg? | 1. Yes 2. No |
| Question 10 | When did you first actively come into contact with the robot? | 1. Never 2. Before residency (e.g. as ANIOS or researcher) 3. Year 1 4. Year 2 5. Year 3 6. Year 4 7. Year 5 8. Year 6 |
| Question 11 | Did you use the Skills Simulator before your first bed-side assistant procedure? | 1. N.a. 2. Yes 3. No |
| Question 12 | Did you use the Skills Simulator after your first bed-side assistant procedure? | 1. N.a. 2. Yes 3. No |
| Question 13 | Did you use the Skills Simulator before your first console procedure? | 1. Yes 2. No |
| Question 14 | Did you use the Skills Simulator after your first console procedure? | 1. N.a. 2. Yes 3. No |
| Question 15 | In which year of residency do you think robotic-assisted surgical training would ideally start (including simulation)? | 1. Year 1 2. Year 2 3. Year 3 4. Year 4 5. Year 5 6. Year 6 7. Fellowship |
| Question 16 | Would you like to participate in national robotic-assisted surgical training? | 1. Yes 2. No, already completed 3. No, not interested |
| Question 17 | How long ago did you have your first active experience with the robot? | 1. Never 2. 0-6 months 3. 6-12 months 4. 1-5 years |
| Question 18 | How many procedures have you assisted as bedside-assistant? | 1. Never 2. 1-10 procedures 3. 10-50 procedures 4. >50 procedures |
| Question 19 | How many procedures have you performed on the robot as a (partial) console surgeon? | 1. Never 2. 1-10 procedures 3. 10-50 procedures 4. >50 procedures |
| Question 20 | How many procedures have you performed on the robot as first console surgeon? | 1. Never 2. 1-10 procedures 3. 10-50 procedures 4. >50 procedures |
| Question 21 | How important do you think robotic-assisted surgery will be in the future? | 1. Robotic-assisted surgery will disappear in the future 2. Robotic-assisted surgery will be used selectively next to laparoscopy, in the future 3. Robotic-assisted surgery will take over most laparoscopy in the future 4. Robotic-assisted surgery will become the new ‘standard’ in the future |
| Question 22 | How important do you think robotic-assisted surgery will become for your own future? | 1. I will not use robotic-assisted surgery in my future 2. I will use robotic-assisted surgery selectively, next to laparoscopy, in my future 3. Robotic-assisted surgery will play a big role, but will not overtake all laparoscopy in my future 4. Robotic-assisted surgery will be my new ‘standard’ in my future |
| Question 23 | Do you think you will work on the robot yourself during residency? | 1. Yes, plenty 2. Yes, but limited 3. No, probably not 4. No, not at all |
| Question 24 | Do you think that, after graduating as a surgeon, you will work on the robot yourself in the first 3 years after residency? | 1. Yes, plenty 2. Yes, but limited 3. No, probably not 4. No, not at all |
