# Supplementary material for: Exploring needs, prevalence and experience with robotic-assisted surgery training among residents: a mixed method study
Source: J Robot Surg. 2025 Jul 15;19(1):392. doi: 10.1007/s11701-025-02527-7 (PMC12263794; doi:10.1007/s11701-025-02527-7)
Supplement: Supplementary file 2 — Supplementary file2 (DOCX 14 KB) [file 11701_2025_2527_MOESM2_ESM.docx]

**Supplementary Table 2** Interview guide in Dutch, used for the focus group interviews

|  | **Question** |
| --- | --- |
| Question 1 | Could you introduce yourself to the group by indicating the current year of your residency program, specifying whether it is within an academic or peripheral hospital? Additionally, please provide details regarding the current level of experience with robot-assisted surgery and your training in this field. |
| Question 2 | Could you describe your ideal training program? |
| *In-depth questions* | What type of training?  Non-technical skills?  Who should conduct the training? |
| Question 3 | For which residents is this training suitable? |
| *In-depth questions* | In which year would you prefer to commence your training?  Should residents have to apply for this training? |
| Question 4 | Should there be an assessment or certification tied to the course? |
| *In-depth questions* | Who should conduct the assessment?  Should we use OSATS or specific R-OSATS?  Is participation sufficient for a certification?  Role of the industry? |
| Question 5 | If training is provided in your hospital or by the program director, what aspects of this training could be improved? |
|  | In what way has robotic-assisted training contributed to your development as urologist/surgeon/gynecologist? |
| Question 6 | What are your expectations for robotic-assisted surgery and its training? |
